# From global-to-local: rural mental health in South Africa

**DOI:** 10.1080/16549716.2017.1413916

**Published:** 2018-01-11

**Authors:** Richard Vergunst

**Affiliations:** Department of Psychology, Stellenbosch University, Matieland, South Africa

**Keywords:** Rural, mental health, South Africa

## Abstract

In this paper, the current situation regarding rural mental health in South Africa is explored. The current status is presented, followed by an attempt to provide approaches and ideas to improve the situation in order to make it more context appropriate and relevant. Issues of staffing, task shifting or sharing, and formal vs informal health care systems are considered and discussed as possible future approaches to improve rural mental health care in South Africa.

## Background

Global mental health aims to address the inequities in mental health between low-income and high-income countries [1]. Mental health is a neglected priority in many low- and middle-income countries (LMIC) [2], while rural areas in these countries seem to be even further neglected. This paper explores the status of rural mental health in South Africa and attempts to propose effective ideas and approaches for the future so that mental health care and services are accessible to all in South Africa – both urban and rural. The first part of the paper will be an attempt to understand and define rural health before contextualising how rural mental health fits into the rural health of South Africa.

## Rural health

Rural health has generally been a relatively neglected area of health research. Interest has, however, grown in the past several years [3]. In the past, rural health was generally seen as a ‘vague idea’ that basically looked at medical work outside of mainstream urban areas, but is now becoming more well-known and better understood [4]. The conceptualisation and measurement of the construct is becoming increasingly important to research and policy in the field of rural health [5].

## Defining rural health

A minimum of three primary domains have historically been central to the definition of what is rural. These are the ecological, occupational and sociocultural components [6]. The ecological component refers to the spatial apportionment of the population. This is conventionally employed to signify a delimited geographical area characterised by a population that is small, relatively sparse, and isolated, to varying degrees, from metropolitan hubs. The occupational dimension is probably the most well-defined, referring to people who get their income from agriculture, mining, fishing, forestry, etc. The sociocultural dimension of rurality is the most complicated, but generally refers to values and ideals that underlie human interactions in a rural setting.

There is, however, no clear specific definition of what is meant by ‘rural’. It is a ‘theoretically rich’ construct [5, p. 5] and has a multidimensional character in terms of its conceptualisation and measurement [7,8]. There is no consensus on the definition of what constitutes a rural area and there are many definitions of the term [3]. It is difficult to reach agreement about the definition of rural [9]. There is a need in the future for universal definitions so that we can compare studies and carry out future collaborative research [10]. Muula [3], however, states that it is not possible to have a universal definition. The key is what purpose the term is used for [9]. In most instances authors assume readers have specific knowledge on what is being referred to as rural. Most definitions, according to Couper [9], take issues such as service, access and distance into account. The way that rural health in the South African context is understood, addresses issues of poverty and inequity and hence has a strong social justice, social responsibility and advocacy component, going beyond the common technicalities of geography and distance [4]. In South Africa there is no standardised definition of rurality, and various stakeholders use a variety of criteria to define rural – or do not use rural as a variable at all [11].

## Rural health in South Africa

Rural health care practice, like virtually every other activity in South Africa, has been deeply shaped and impacted on by the political situation in the country over the last 50 years [12] and more. Rural health in South Africa is synonymous with the health of the deliberately underdeveloped areas of the country, inhabited largely by Black communities. Since the election in 1994, there have been plans for sweeping changes to the health care system and the priority principle of the plan was that of equity. Equity has direct implications for rural health care and practice in South Africa. Now the quality of rural health care services can be seen as a barometer of success of the broader social reforms undertaken by the government. The South African government has prioritised the needs of persons living in rural areas, with increasing attention being paid to social and economic development [13].

In South Africa, 52% of the total population and 75% of poor South Africans live in rural areas [4]. South African society is a society in transition and this is reflected in its morbidity, mortality and disability profiles [12]. The health status of rural people in South Africa is similar to that of people in many developing nations around the world. The diseases of poverty are common, including chronic disability. Access to health care for rural people is difficult: the high cost of transport and the large distances involved lead to late presentations of disease. This is further complicated by traditional beliefs regarding illness; unregulated traditional healers of various levels of experience and skill make their services available to a somewhat fearful and tradition-bound public in rural areas [12]. According to Gaede and Versteeg [11, p. 99], rural communities in South Africa experience ‘significant barriers to accessing healthcare’, including financial barriers, inadequate transport, and distances to the nearest facility as well as limited resource availability.

The public health care system in rural areas has been delivered through a system of rural hospitals and clinics, many of which were built and operated as mission hospitals until the 1970s, when most of them were taken over by the apartheid government in an effort to centralise planning. These same hospitals now form the infrastructure for the new National Health System, the aim of which is to decentralise to a district-based health system. The infrastructure and facilities available in rural hospitals are relatively good, although diagnostic services are limited. Most rural hospitals offer a comprehensive service where doctors with general training are employed and who are largely foreign-qualified [4].

How an elderly woman with disability living in a rural area can access quality health care will act as a barometer of South Africa’s progress towards a more just, fair and civilised society, until a more objective measurement is developed [4]. Little research has been conducted into the experiences, needs and challenges of those living in rural areas in South Africa, particularly among persons with disabilities [14] – especially when it comes to psychosocial disabilities or mental health. Rural mental health is not well integrated into rural health care in South Africa. As Ms Ingrid Daniels, director of the Cape Mental Health Society in South Africa, stated at a rural conference in 2014, the provision of mental health services in South Africa is fragmented, limited or non-existent, particularly in remote rural areas. This statement is based on the experiences of mental health societies’ narrative reports across the country.

## Rural mental health in South Africa

There is a lack of mental health care services in rural South Africa. This situation has been labelled as ‘dehumanising’ by the South African Rural Mental Health Campaign Report published in 2015 [15]. According to the report, rural areas account for almost half the country’s population but still remain the most underserved and marginalised.

For example, there is a disproportionate distribution of mental health human resources in urban and rural areas within South Africa. For instance, based on some data, the density of psychiatrists in or around the largest city is 3.6 times greater than the density of psychiatrists in the entire country. The distribution of mental health nurses between urban and rural areas is not known [16]. Rural areas in South Africa often do not have psychiatrists or psychologists and rely primarily on general doctors, occupational therapists and nurses for mental health interventions. Should someone need a psychologist or psychiatrist, they are often referred to the nearest city which sometimes involves transport issues.

The implication is that the internationally acclaimed South African state-of-the art, rights-based Mental Health Care Act is a good example of a health policy which aims at improving access to mental health care at local level but which is difficult to implement in rural areas [17].

Burgess [18, p. 735] found that the implementation of the primary mental health care model in a rural area in South Africa was ‘fraught with complications’. For example, mental health service users did not enter the services through outlined pathways for screening and referral. Also, existing staff shortages, combined with inadequacies in dealing with patients, resulted in use of physical or pharmacological restraints. Further, general nurses had limited engagement with patients and there were high turnovers of community service workers.

De Kock and Pillay’s [19, p. 7] situational analysis of mental health nurses’ resources in South Africa suggests a ‘distressing shortage’ of these nurses in South Africa’s rural areas. According to their situational analysis, the lack of medical officers who are able to prescribe medication in rural areas has meant that this task has been shifted to mental health nurses. But only 62 (38.7%) of 160 facilities employ mental health nurses, a total of 116 mental health nurses. These nurses serve more than 17 million people, indicating that mental health nurses are employed at a rate of 0.68 per 100 000 population in South Africa’s rural areas. This, according to them, is a ‘most dire’ situation (19,p. 1). This with the backdrop that mental health human resources have generally been a challenge, with South Africa having lower workforce numbers than many other LMIC [20]. It is within the rural areas that these human resource challenges are the greatest [21].

Despite these problematic issues, there has been a paucity of research looking at rural mental health care in South Africa. Studies [22,23] have confirmed that pathways to mental health care in rural South Africa are complex. These pathways can include formal western medicine as well as informal traditional medicine, with over half of the cases reporting no contact with formal health care services. This highlights the important role of informal care providers for rural mental health in South Africa and that interventions beyond the formal health services need to be looked at as an alternative. Hence, Kirmayer and Pedersen [1] advocate that there is a need for interventions beyond the formal health services and a shift towards more community-based approaches, including self-help and peer-support. What is required is a more open and creative approach in proposing alternatives (such as informal health services as well as community-based services) to mental health intervention, particularly in rural areas.

Two doctoral studies by Braathen [24] and Brooke-Sumner [25] investigated the state of rural mental health in South Africa. Braathen’s [24] findings revealed, in part, that there needs to be a shift of care from pacifying to activating – and that this needs to be broader than health care. Also, care for people with mental illness is not just about cure and treatment, but is also about prevention and promotion of mental health. Hence, a pluralistic and holistic approach to care is most appropriate where a combination of scientific medicine, community-based approaches, traditional health providers and structures outside health care systems including religious societies, NGOs, families and the broader community all have a role in rural mental health care in South Africa. This is supported by Havenaar et al. [26] who mention that traditional healers must play an integral role in the country’s mental health care system.

Brooke-Sumner [25] found that a task-shared model of intervention was acceptable and feasible. The concept of task sharing or shifting refers to the use of non-specialist health care workers in providing services to users under the supervision of specialists. These non-specialists can include general nurses, community health workers, traditional healers and spiritual leaders. She, however, warns that there is a crucial need for allocation of further resources given that no mental illness rehabilitation service delivery platform exists in many areas in South Africa. This task sharing or task shifting perspective for rural mental health is supported when Petersen et al. [27] state that rural areas in South Africa are particularly under-serviced when it comes to psycho-social rehabilitation programmes, and conclude that the adoption of task shifting can close mental health service gaps in rural areas.

However, the idea of task shifting or sharing is not always easy to implement. A study by Petersen et al. [28] suggests that a lack of training and support from mental health specialists impeded primary health care nurses’ capacity to provide mental health care in rural areas. Part of the problem is that the training of mental health specialists (psychiatrists and psychologists) in South Africa is primarily focussed on individual perspectives and not on community perspectives. More appropriate training at tertiary educational institutions in South Africa, that is more relevant to rural dynamics, is required to address the gaps in service delivery in these regions. These specialists will then be better equipped to train nurses, community health workers, occupational therapists, traditional healers, etc., who work in rural areas. There needs to ‘be more efficient use of existing resources’ [28, p. 140] but this can only be done if there is adequate training, supervision and support [29].

In order to provide appropriate mental health care in rural areas in South Africa, we need to take a broader perspective than the formal health care system alternatives and include other intervention strategies that are context appropriate [30]. This is because interventions that are not ‘locally relevant and culturally consonant’ could lead to negative outcomes including inappropriate diagnoses and interventions which could lead to increased stigma and poorer health outcomes [1, p. 759]. For instance, more community participation in the delivery of mental health care may be more appropriate in deep rural areas where there are shortages of western-trained medical personnel and where the culture shapes illness experience [1]. The experience of mental illness is filtered by complex cultural belief systems, with culture-bound syndromes as well as culture specific events influencing the understanding and treatment of them [30]. This notion is supported by Musyimi et al. [31] who recommend that a relationship between informal (faith and traditional healers) and formal (clinicians) needs to be based on trust and respect in order to improve mental health care in rural areas.

The question is if these varying stakeholders (formal and informal) in mental health care are able to work alongside each other in order to optimise mental health care in rural areas in South Africa. According to Campbell-Hall et al. [32], one needs to build respectful collaborative relationships in the interest of improved patient care. However, most informal healers are willing to refer patients to formal health care settings, but this is not reciprocated [33,34]. Despite this, Gureje et al. [35] state that there are possibilities for collaboration between varying stakeholders in the care of persons with mental illness, but Musyimi et al. [31] mention that referral systems between these stakeholders are weak and need to be strengthened. Working relationships can only enhance mental health care in rural areas in South Africa, but mechanisms to strengthen referral systems seem to be lacking. For Gureje et al. [35], research is still required to clearly delineate the boundaries of such collaboration and to test the effectiveness in treating mental illness in different contexts. Burns and Tomita [36] have proposed recommendations to include innovative programmes to foster collaboration between the different stakeholders in order to improve mental health care in Africa. As Musyimi et al. [31, p. 7] conclude:

It is necessary for traditional healers, faith healers and clinicians to continuously engage in respectful dialogue and open two-way dialogue to understand each other and build mutual respect and trust to move forward with medical pluralism and increase mental health outcomes in patients.

The ‘revolving door’ phenomena where two-thirds of patients with mental illness in a rural area in South Africa were re-hospitalised within one-year follow-up is also an issue that needs to be addressed [37]. Again, this highlights that possible task shifting or sharing, as well as using more informal sources of intervention, may be more appropriate than following the classical care pathway of hospital admission within the western biomedical model. The *context* of the person and his/her mental health needs to be taken more seriously in rural areas in South Africa – a wider perspective in terms of understanding culture, aetiology and management could play a more important role in their care than depending on western medical care alone.

Western care with medication may not always be the most effective intervention for mental illness. Read [38, p. 438] explored the limits of anti-psychotic medication in rural Ghana where her findings suggest that, in order to improve the treatment of mental illness in rural areas, one should take into account the limitations of antipsychotic drugs and consider how ‘local resources and concepts of recovery can be used to maximise treatment and support families’. Exclusive attention to mental illness through the psychiatric lens may not focus on the social structural determinants of mental health that may be more appropriate in certain contexts [1]. This is where interventions outside the biomedical model may be more appropriate and effective. There is this tension between biomedical models and socially and culturally informed community-based approaches that focus on social determinants of mental health and the need to listen to local issues [39]. This tension needs to be addressed so that the different models and approaches can work together for the benefit, not only of the individual, but also the family and community.

## Conclusion

Rural mental health is complex, with many ramifications and implications for those involved in it in one way or another [40]. The literature on rural mental health in South Africa is sparse but increasing as this becomes an important area of mental health within the country. In the past, it has been a relatively neglected area of interest. However, the fact that it is growing in interest and gaining more momentum does not necessarily mean that it is in a healthy state of existence. Despite good intentions in policy, there are still many implementation obstacles and challenges.

This paper has shown that one cannot and should not depend on the western biomedical model alone in attempting to address mental health in rural areas in South Africa in particular. This is due to a number of reasons, including a lack of appropriately trained medical staff. Task shifting or sharing may be a very feasible option to overcome this burden. Likewise, the inclusion of other informal stakeholders in the care programme of mental health service users (such as traditional healers, community health workers, religious organisations, families, self-help groups) may bridge the gap to mental health programmes. In other words, we need to think creatively when it comes to effective rural mental health intervention programmes. These intervention programmes all need to be locally relevant to the specific rural contexts. One cannot only offer a narrow biomedical approach to rural mental health in South Africa – especially where indigenous, cultural or local systems play a vital role in the aetiology and understanding of mental illness. As Kirmayer and Swartz [41, p. 41] succinctly summarise, global mental health tends to emphasise professional mental health interventions and may marginalise indigenous forms of helping, healing, and social integration that can contribute to positive outcomes and recovery.

## Paper context

There is not much known or discussed about the current status and dynamics of rural mental health in South Africa. This paper hopes to set rural mental health in South Africa in context and create a better understanding of what it is about. The implication is that a conversation around this underrepresented area of health in South Africa will be initiated so that more can be done to improve this area of health.
